# Impact of Two Brown Seaweed (*Ascophyllum nodosum* L.) Biostimulants on the Quantity and Quality of Yield in Cucumber (*Cucumis sativus* L.)

**DOI:** 10.3390/foods13030401

**Published:** 2024-01-26

**Authors:** Tilen Zamljen, Helena Šircelj, Robert Veberič, Metka Hudina, Ana Slatnar

**Affiliations:** Department of Agronomy, Biotechnical Faculty, University of Ljubljana, Jamnikarjeva 101, SI-1000 Ljubljana, Slovenia; helena.sircelj@bf.uni-lj.si (H.Š.); robert.veberic@bf.uni-lj.si (R.V.); ana.slatnar@bf.uni-lj.si (A.S.)

**Keywords:** algae extract, biostimulation, metabolic response, plant growth, yield, food

## Abstract

Algal biostimulants are increasingly integral to vegetable cultivation due to their capacity to boost yield, alleviate abiotic and biotic stress, and enhance overall crop quality. This study evaluated the impact of two commercially available algal-based biostimulants on cucumber (*Cucumis sativus* L.), examining their effects on yield, number of fruits, dry weight, color, flesh thickness, skin thickness, plastid pigments, and tocopherol content. Both biostimulant treatments resulted in a roughly 13% decrease in yield and fruit number compared to the control treatment. Notably, the biostimulants positively influenced the fruit brightness parameter (L*), leading to darker fruits. Fitostim^®^ algal biostimulant exhibited a positive effect on dry weight during the initial harvest. The predominant pigments were chlorophyll a and chlorophyll b (constituting 80% of all analyzed pigments), and the most abundant tocopherol was α-tocopherol, comprising 80% to 90% of tocopherols. Skin tissues contained significantly higher levels of pigments and tocopherols compared to flesh. Both biostimulants caused a notable decrease in total tocopherol content in the skin at the first harvest, with reductions of 19.91 mg/kg DW for Phylgreen^®^ and 9.43 mg/kg DW for Fitostim^®^ algae. The study underscores the variable efficacy of biostimulants, emphasizing their dependence on the specific biostimulant type and fruit part. The application of biostimulants has the potential to substantially enhance the internal quality of cucumbers, particularly in terms of plastid pigments and tocopherols, offering potential health benefits for consumers.

## 1. Introduction

Cucumber (*Cucumis sativus* L.) is a common vegetable that belongs to the Cucurbitaceae family, which consists of more than 800 species [1]. Worldwide, cucumber is the fourth most cultivated vegetable, after tomato, cabbage and onion [2]. Cucumber is a frost-sensitive vegetable that requires temperatures above 15 °C for normal growth and development and it requires large amounts of water and fertilizer to produce adequate yields [1]. Fruits often develop partenocarpically. Depending on the cucumber variety, yields can range from 2 to 20 kg per m^2^ [3]. Additionally, it is one of the more sensitive plants to pests and disease, so usage of plant protection substances is common. In recent years, a more sustainable approach has been considered by the European Union, prompting the use of alternative substances such as biostimulants in cucumber production [2,3].

Biostimulants are widely used in agriculture to compensate for negative environmental factors and to improve the quantity and quality of yields. There are different types of biostimulants such as microbial inoculants, humic substances such as humic acids and fulvic acids, protein hydrolysates or amino acids, biopolymers, inorganic compounds, and algal extracts [4].

Algal extracts are a common and widely used biostimulant based on marine algae (*Ascophyllum nodosum* and *Ecklonia maxima* are the most commonly used). Several factors influence the composition of algal biostimulants, such as the species of algae used, the source of the algae, the time of collection, and the extraction method [5]. The preparation of algae-based biostimulants can be accomplished by different methods, such as physical (heat, pressure, and microwaves) and chemical methods (solvents, acids, and alkalis). The choice of extraction method for marine algae determines how complex the resulting biostimulant will be and ensures sufficient integrity of the biologically active molecules that act as biostimulants [6]. Usually, the extraction is performed under alkaline conditions and high pressure. The algae extracts are usually cold-pressed. Although this method lowers the concentration of some molecules (especially certain hormones), high concentrations of oligomers, which are among the most biologically active components of algae, are obtained [7], which can influence yield. Algal extracts are a complex mixture of macro- and micronutrients and metabolites, so studies on the exact function of algae-based biostimulants are very complex [6]. Usually, the biostimulant acts on several different metabolic pathways and plant functions simultaneously, making it difficult to determine the exact functional principle in the treated plant [8]. They stimulate seed and seedling germination and growth, increase yield, flowering, and fruit set, and reduce the negative effects of biotic and abiotic stress. There are also reports that algal extracts improve shelf life and postharvest quality of vegetables [9]. Algal extracts have an impact on plants’ primary and secondary metabolism as reported by Zamljen, Hudina, Veberič and Slatnar [8]. Cucumbers contain several groups of primary and secondary metabolites such as tocopherols and plastid pigments.

Tocopherols are synthesized exclusively by organisms that perform photosynthesis. There are four main tocopherols, namely α-, β-, γ-, and δ-tocopherol [10]. The most abundant tocopherols are α- and γ-tocopherol. α-Tocopherol accumulates mainly in photosynthetic tissues. Tocopherols are important for scavenging reactive oxygen species (ROS) in plants [11]. They also have beneficial effects on human health, such as antioxidant and anti-inflammatory effects [12].

Plastid pigments are among the most important metabolites for plants because they are responsible for the photosynthetic activity of plants and absorb light in the visible range [13]. The plastid pigments contain chlorophyll and carotenoids. Chlorophyll compounds are mainly chlorophyll a and chlorophyll b, which are the majority of chlorophylls [14]. Carotenoids are the most abundant and widespread pigment group in nature. The most important carotenoids are β-carotene, lutein, violaxanthin and neoxanthin [15].

Based on the various reported effects of algal extracts on fruits and vegetables, we conducted a study to investigate how two algal-based biostimulants affect cucumber fruit parameters, yield quality and quantity. Our study was carried out in normal growing conditions at full scale and not at laboratory scale. To add additional depth to our study, total and individual pigments and tocopherols in the skin and flesh of the cucumber were analyzed, due to the positive effects on human health and importance for human consumption. Additionally, the total and individual pigments and tocopherols have previously, to the best of our knowledge, been poorly studied in cucumbers treated with biostimulants.

## 2. Materials and Methods

**Figure 1:**
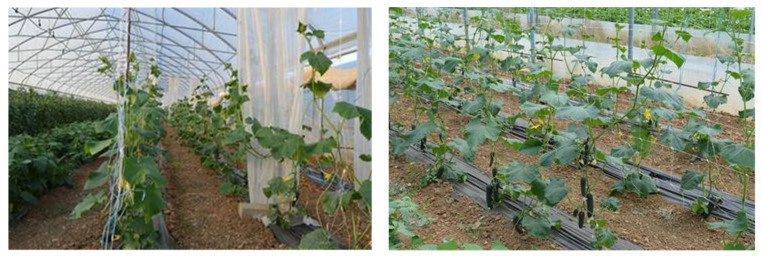
Cucumber in the experiment, separated by a plastic net.

The experiment was conducted in 2018 at the Biotechnical Faculty Horticultural center in Orehovlje, Slovenia (45°53′13.91″ N; 13°36′37.84″ E). Cucumber (*Cucumis sativus* L.) ‘Lisboa F1’ plants were planted in a plastic greenhouse on 5 April 2018, after 2 to 3 true leaves appeared (approximately 6 weeks after seeding). The experiment was conducted for 116 days, until 30 July 2018. The plants were planted at a distance of 60 cm between plants and 100 cm between rows. A plastic net was attached to the structure of the greenhouse to serve as a support for the plants (Figure 1). The cucumbers were planted on black plastic foil, under which an irrigation hose was placed.

**Figure 2:**
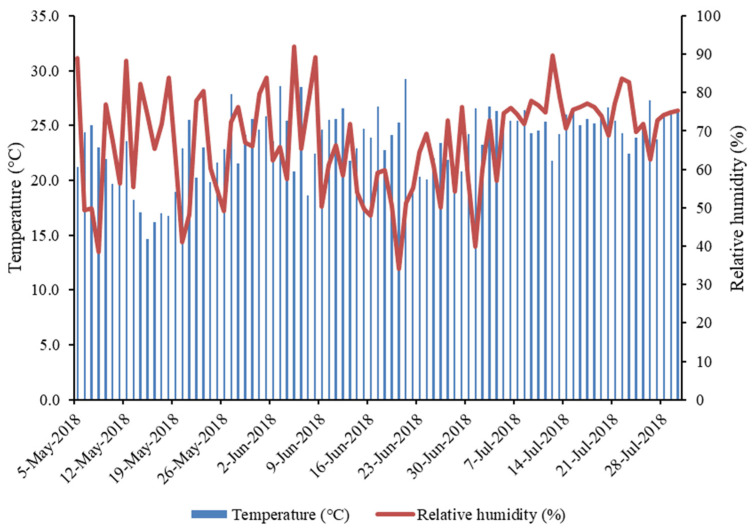
Temperature (°C) and relative humidity (%) in the greenhouse during the experiment period. Red arrows show the three main harvest dates for chemical analysis.

The cucumbers were irrigated daily with a drip irrigation system, with the amount of irrigation determined depending on the stage of development and the temperature in the greenhouse (Figure 2). The monthly hours of sunshine during the experimental period were 215.5 h (April), 228.9 h (May), 267.5 h (June), and 271 h (July). Cucumber plants were optimally irrigated trough out the growth season. A barrier of plastic film was placed between treatments to ensure that the biostimulant was applied only to the correct treatment. Cucumbers were treated with three treatments: (i) control treatment with regular water; (ii) treatment with algal biostimulant Phylgreen^®^; and (iii) treatment with algal biostimulant Fitostim^®^ algae.

The experiment was designed in a randomized block design. Each of the three blocks contained 25 plants of each treatment (75 plants for each treatment). Each block was 15 m^2^ in size (Table 1).

The pest and disease control during the growth period was maintained based on the guidelines for the integrated production of vegetables.

The biostimulants were first applied foliarly 5 days after transplanting into the greenhouse, and then the cucumbers were treated every 10 days until the end of the study. The concentration of biostimulants was 0.3%, as recommended by the manufacturer.

The algae-based biostimulant Phylgreen^®^ (Trade Corporation International, Madrid, Spain) is a brown algae extract obtained by cold-pressing from *A. nodosum* L.: 15% dry matter, 0.2% N, 0.05% P_2_O_5_, 0.4% K_2_O, 8% C, and 1.2% mannitol. The pH ranged from 3.5 to 4.5 and the density was 1.1 kg per liter. This biostimulant can be applied foliarly or into the soil weekly, with a concentration of 0.15% to 0.30%, depending on the plant species and development stage.

The algae-based biostimulant Fitostim^®^ Algae (SCAM SpA, Modena, Italy) is a brown algae extract from *A. nodosum* L. The technical specifications were as follows: 2% N, 10% C, and 50% organic compounds with molecular weight greater than 50 kDa. The pH ranged from 7.5 to 8.5. It can only be applied foliarly, with a concentration of 0.2% to 0.3%. Both producers suggest a 7- to 10-day application during the growth period.

### 2.1. Fruit Sampling

Fruit was harvested every 2 to 3 days for a total of 80 days (with the first harvest on 11 May), when they reached sufficient size (18–20 cm in length). Yield and number of fruit were recorded at each harvest. For laboratory analysis, fruits were harvested on 8 June, 20 June, and 4 July (Figure 2). Most fruit was harvested on these three harvest dates. From each block, 5 fully developed fruits were randomly picked for each treatment. In total, each treatment was represented by 15 fruits on each harvest date. Fruits were scored for skin color: L* (lightness), a* (redness: green to red), b* (yellowing: blue to yellow), using a non-destructive Konica Minolta portable colorimeter (CR-10 Chroma, Tokyo, Japan). For the dry matter, the samples were weighed fresh and then placed into an oven at 105 °C for 48 h or until stable mass. After drying, they were weighed again. Placenta and flesh diameter were measured using a digital beak scale. The fruits were then peeled using a fruit peeler and instantly frozen in liquid nitrogen. The cucumber flesh and skin were lyophilized, and stored at −20 °C on all harvest dates for chemical analysis for pigments and tocopherols.

### 2.2. Extraction and Determination of Plastid Pigments and Tocopherols

The extraction and determination of pigments and tocopherols was based on Schmitzer et al. [16]. In brief, 0.2 g of lyophilized sample was extracted with 2 mL of cold 100% acetone under dimmed light.

The samples were analyzed on a Survey HPLC system (Thermo Finnigan, San Jose, CA, USA). For the pigments’ determination, a diode array detector was used at a wavelength of 440 nm, coupled with a Spherisorb S5 ODS-2250  ×  4.6 mm column (Allbtech Associates, Inc., Deerfield, IL, USA). For the pigments, the mobile phase A was acetonitrile, water, and methanol (100:10:5; *v*/*v*/*v*) and mobile phase B was acetone with ethyl acetate (2:1; *v*/*v*).

For the determination of tocopherols, a Spherisorb S5 ODS-2250  ×  4.6 mm column on Spectra-Physics HPLC system with FL 2000 detector (Fremont, CA, USA) set at excitation 295 nm and emission 325 nm was used. Acetone extracts were subjected to isocratic HPLC analysis, with methanol as solvent. The contents of pigments and tocopherols were determined using external standards. The data were expressed as mg/kg dry weight (DW).

### 2.3. Chemicals

α-, β-carotene, chlorophyll a and b, lutein, neoxanthin, violaxanthin, antheraxanthin, and zeaxanthin were from DHI LAB products (Hoersholm, Denmark), and α- and γ-tocopherols from Sigma-Aldrich (Steinheim, Germany).

### 2.4. Statistical Analysis

The R program [17] was used for statistical analysis. All data are expressed as mean ± standard error (SE). To determine whether differences were observed between treatments, a one-way analysis of variance (ANOVA) with LSD test was used. The confidence level was 95%. A correlation analysis was performed among different variables using the Pearson’s test for correlation (α < 0.05).

## 3. Results

### 3.1. Yield of Cucumbers

**Figure 3:**
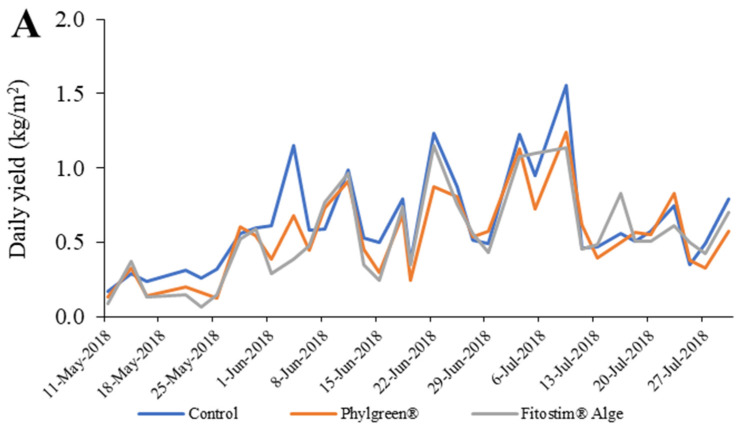

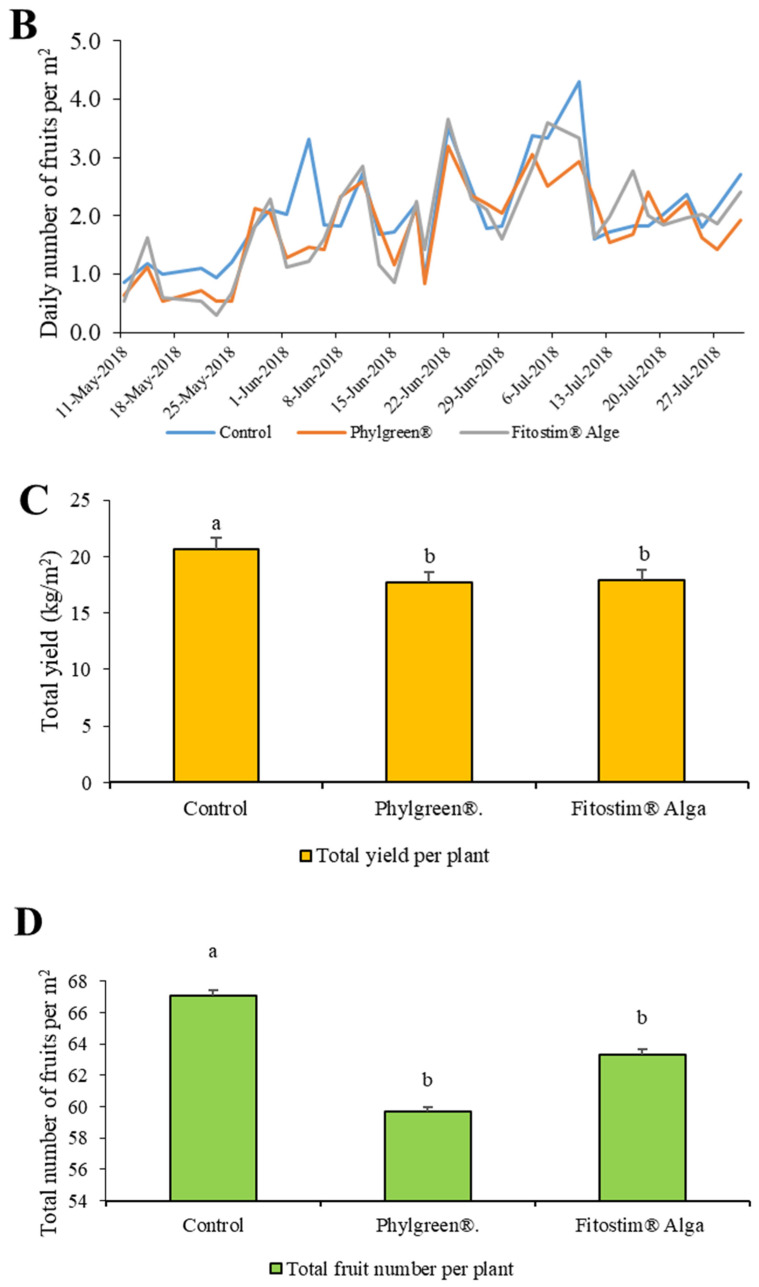
Daily yield (**A**), daily number of fruits (**B**), total yield (kg) (**C**), and total number of fruits (**D**) for cucumbers treated with algae biostimulants. Data are means ± standard error (n = 5). Data with different lowercase letters (a to b) are significantly different (HSD test; *p* < 0.05).

Yield and number of fruits were assessed daily from 11 May to 27 July, as shown in Figure 3A,B. The daily yield was low in the first 24 days (until 4 June), which was due to the intense growth of cucumbers. The daily yield of cucumbers increased rapidly from 4 June to 11 July, and we observed three peaks in which we harvested up to 1.5 kg of cucumbers per m^2^. After this period, the yield decreased. A similar pattern was observed for the daily number of fruits per m^2^, with up to 4.5 cucumbers harvested per m^2^ at the peak of the cucumber harvest.

Total yield and fruit number were highest in the control treatment (Figure 3C,D), with 20.6 kg and 67.1 cucumbers harvested per m^2^, respectively. Total yield per m2 and fruit number per m^2^ were lower in both biostimulant treatments. In the Fitostim^®^-Alga treatment, the total yield was 17.8 kg/m^2^ and the total number of fruits per m^2^ was 63.3. The Phylgreen^®^ treatment had a total yield of 17.7 kg/m^2^ and a fruit count of 59.7 per m^2^. Correlation analysis showed that yield and number of fruits were strongly correlated with harvest date and less correlated with biostimulant treatment (Table 2).

### 3.2. Fruit Parameters

Skin color, dry weight, diameter of the flesh and placenta are shown in Table 3. Skin color parameters (a*, b*, L*) varied less among treatments on each harvest date than among all three harvest dates. The color parameter a* showed that the cucumbers treated with Phylgreen^®^ were less green at the first harvest than the other two treatments. On the other hand, the control cucumbers were lighter in color (L*) than the cucumbers treated with algae at the first and third harvests. Dry weight was positively affected by Fitostim^®^ algae by 5.2% in the first harvest, compared to the control and Phylgreen^®^ treatments. No differences were observed between treatments in the other two harvests. The diameter of the flesh and placenta showed no differences between treatments.

In the total statistical analysis of each parameter, there were no significant differences between dry weight, flesh diameter, and placenta diameter among treatments. The a* parameter showed that the Phylgreen^®^-treated fruits were less green, and the L* showed that the fruits were darker compared to the other two treatments. The color of fruits was strongly correlated to the fruit part (R^2^ = 96.3), biostimulant treatment (R^2^ = 89.6), and total pigment contents (R^2^ = 98.6) (Table 2).

### 3.3. Plastid Pigments and Tocopherols in Cucumber

#### 3.3.1. Plastid Pigments and Tocopherols in Cucumber Flesh

**Figure 4:**
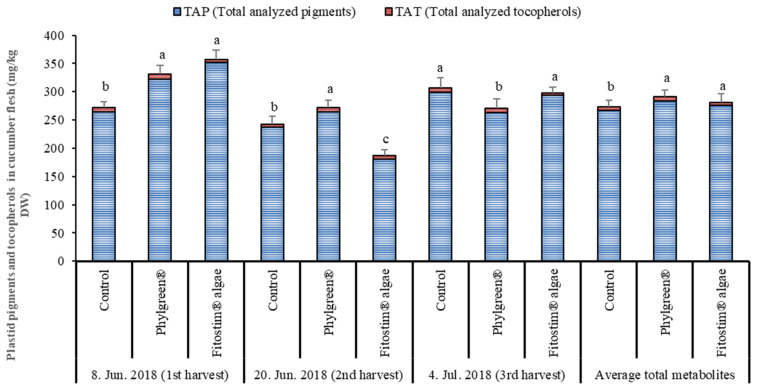
Total pigments and tocopherols in cucumber flesh picked at three harvest times. Data are means ± standard error (n = 5). Data with different lowercase letters (a to c) for each individual substance are significantly different among treatments and within the same harvest dates (HSD test; *p* < 0.05). TAP = total analyzed pigments; TAT = total analyzed tocopherols.

The contents of individual and total pigments and tocopherols in the flesh of cucumber are listed in Table S1. The total pigments and tocopherols in cucumber flesh are presented in Figure 4. A total of seven pigments and two tocopherols were identified in the flesh of cucumber. The most abundant pigments were chlorophyll a and chlorophyll b, which accounted for approximately 80% of all pigments analyzed in the flesh. The most abundant carotenoids were lutein and β-carotene. Lutein presented approximately 49% of all determined carotenoids. In the first harvest, both biostimulants had a stimulative effect on the content of individual and total pigments compared to the control treatment. The effect of the biostimulants on pigment content was lower in the later harvests. In the second harvest, the biostimulant Phylgreen^®^ increased the total content of analyzed pigments by 26.73 mg/kg DW (+10.1%) compared to the control treatment. On the other hand, the Fitostim^®^ alga decreased the pigment contents in the second harvest compared to the control. Interestingly, the biostimulant Phylgreen^®^ decreased the individual and total pigments at the third harvest compared to the other two treatments. Fitostim^®^ algae had a lesser effect on total tocopherol content, and Phylgreen^®^ biostimulant had a stimulative effect compared to the control treatment. The stimulative effect of Phylgreen^®^ was evident in the first two harvests, with increased total tocopherol contents of 10.2% and 35.6% in the first and second harvests, respectively. Correlation analysis showed that there was no direct relationship between harvest date and total pigments and total tocopherols analyzed (R^2^ = 1.2 and R^2^ = 14.2, respectively) (Table 2).

#### 3.3.2. Plastid Pigments and Tocopherols in Cucumber Skin

**Figure 5:**
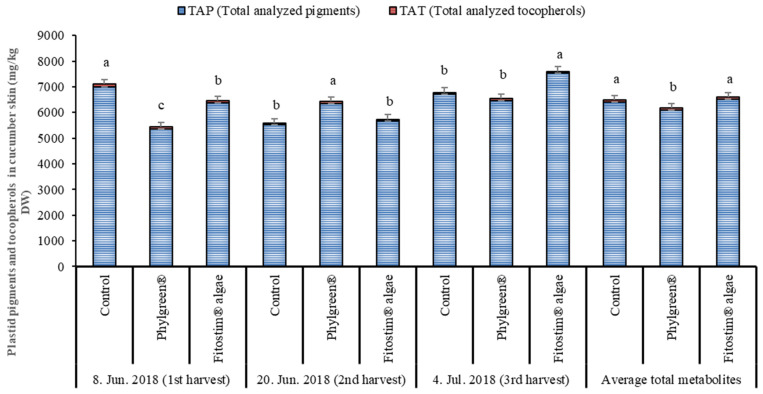
Total pigments and tocopherols in cucumber skin picked at three harvest times. Data are means ± standard error (n = 5). Data with different lowercase letters (a to c) for each individual substance are significantly different among treatments and within the same harvest dates (HSD test; *p* < 0.05). TAP = total analyzed pigments; TAT = total analyzed tocopherols.

The cucumber skin contained larger quantities of plastid pigments and tocopherols compared to the flesh (Table S2). The total pigments and tocopherols in cucumber skin are presented in Figure 5. A total of seven pigments and three tocopherols were detected in cucumber skin. The most abundant pigments were chlorophyll a and chlorophyll b, which accounted for approximately 75% of all pigments analyzed in the skin. The most abundant carotenoids were lutein, β-carotene and neoxanthin. As in the flesh, the skin also contained approximately 50% lutein of the total carotenoids. At the first harvest, both biostimulants had a reducing effect on the total pigments analyzed, compared to the control, with a decrease of 23.4% and 8.9% for the Phylgreen^®^ and Fitostim^®^ algae, respectively. In the second harvest, the Phylgreen^®^ biostimulant had a stimulative effect on total pigment content, and in the third harvest, it had the same effect as the Fitostim^®^ algae compared to the control treatment. The greatest effect of the biostimulants (stimulative or reducing) was on chlorophyll a and chlorophyll b content.

The most abundant tocopherol was α-tocopherol, which accounted for between 80% and 90% of all tocopherols analyzed. In the first harvest, a reducing effect of both biostimulants was observed on the total tocopherol content analyzed compared to the control. The decrease was 19.91 mg/kg DW and 9.43 mg/kg DW for Phylgreen^®^ and Fitostim^®^ algae, respectively. Individual and total pigment and tocopherol contents varied among the three harvest dates, with neoxanthin, antheraxanthin, β-carotene and α-tocopherol in the Phylgreen^®^ treatment and β-carotene and total tocopherols in the Fitostim^®^ algae treatment. Correlation analysis (Table 2) showed that there was no direct relationship between harvest date and total pigments and total tocopherols analyzed. The correlation analysis showed that the greater influence on pigment and tocopherol content depended on the fruit part (skin/flesh).

## 4. Discussion

The biostimulatory effect of two algae-based biostimulants was tested on cucumber (*Cucumis sativus* L.) ‘Lisboa F1’. Yield, number of fruits, color, flesh thickness, placental thickness, dry matter, individual and total pigments, and tocopherols were studied. The physical parameters of cucumber were affected differently by the application of biostimulants, resulting in reducing or stimulating effects. The yield and number of fruits were lower in cucumbers treated with biostimulants throughout the season, which is in contrast to the results reported by Hassan et al. [18]. As reported by Vijayanand et al. [19] for tomato and Rathore et al. [20] for soybean, yield was positively affected by algal-based biostimulant treatment, which is contrary to our results. As reported in the study by Francke et al. [21], yield of shallots decreased by about 14%, which is similar to the results of our study.

A similar result to ours, namely that biostimulants have no effect on yield, was reported by Francke et al. [21] for shallots and by Stasio et al. [22] for grapes treated with biostimulants. Due to the complexity of the biostimulant structure and the lack of studies on such cases, there is no clear answer as to why this is. In certain cases, especially when no stress is induced, certain substances in biostimulants could have a non-stimulant effect, which could be a possible explanation for our results. Another possible explanation for the yield decrease in biostimulant-treated cucumbers could be the stimulation of leaf, root, and stem growth, as was the case in the study by Lefi et al. [23], in which algal-based biostimulants improved plant size but yield did not change. The reason for this, according to the authors’ theory, was the hormone content of the biostimulant.

Biostimulant-treated fruits were darker compared to the control, which is more desirable in cucumbers, as reported by Shan et al. [24]. The darker skin color can be correlated to the higher chlorophyll a content, as supported by our results. Similar results were also reported by Tarantino et al. [25] in apricots and by Soppelsa et al. [26] in apples, where biostimulant-treated fruits were darker than control fruits and showed better coloration. Algal-based biostimulants boost plant metabolism and thus improve coloration [24], which is strongly connected to the plastid pigments contents, especially chlorophyll a, which was also reported by Gitau et al. [27].

Dry weight was increased by the algal biostimulant Fitostim^®^ only at the first harvest; otherwise, the effect on dry weight was negligible. Similar results were reported by Hassan, Ashour, Sakai, Zhang, Hassanien, Gaber, and Ammar [18] for cucumber. On the other hand, Cozzolino et al. [28] reported that the dry weight of tomatoes was significantly increased in plants treated with biostimulants, which could be the result of more intense cell division that can be stimulated by biostimulants.

Individual and total pigments as well as tocopherols were greatly affected by biostimulant application, although the effect was variable, and no general conclusions can be drawn. As reported by Abd-Elkader et al. [29], biostimulants affect photosynthetic pigments, especially chlorophyll a and chlorophyll b. In alfalfa, the algal biostimulant improved chlorophyll a and chlorophyll b content by 12% and 16.7%, respectively [30]. In our study, the effect was similar to some extent, although the efficacy of both biostimulants varied, and in some cases, the pigment content was reduced instead of stimulated. The effect of biostimulants does not usually increase metabolite levels directly, but stimulates enzymatic activity, which then leads to higher or lower metabolite levels [8].

The effect of biostimulants on the pigment contents was lower at the second and third harvest dates in the flesh. In the skin, we observed mixed effects on pigment and tocopherol content. Algae extracts are a rich mixture of carbohydrates, plant hormones, brassinosteroids, polyamines, macro- and micronutrients [21]. Due to the complexity of the biostimulant matrix, the exact principle of its function in plants is still largely unknown. In general, biostimulants improve plant growth, tolerance to abiotic and biotic stresses, and plant quality due to better nutrient uptake [31]. However, the effect of biostimulants can also be reduced or absent altogether, as reported for chilies by Zamljen, Hudina, Veberič, and Slatnar [8]. As shown in the study by Gitau, Farkas, Ördög, and Maróti [27], the choice of the right algal species and extraction method is very important for the efficacy of the biostimulant. As reported by Zamljen, Hudina, Veberič and Slatnar [8], the efficacy of the biostimulant depends on the type of biostimulant, the presence or absence of abiotic and biotic stressors, the intensity of the stress, the genotype of the plant, the application time, the application concentration, and the application rate. The correct application of the biostimulant can have a stimulative effect on the internal quality of cucumbers in terms of plastid pigments and tocopherols, which are especially important for human health.

## 5. Conclusions

Two algal-based biostimulants were tested on cucumber to evaluate the effects on yield, fruit number, dry weight, placental diameter, flesh diameter, individual and total plastid pigments, and tocopherols. The efficacy of the biostimulants was variable, with stimulating or reducing effects. The biostimulants had the strongest stimulatory effect at the time of the first main sampling. In general, the effects on yield were negative, but the effects on plastid pigments and tocopherols were better when cucumbers were treated with biostimulants. It can be concluded that the effectiveness of biostimulants depends on the type of biostimulant. Biostimulants have a great influence on the internal quality of cucumbers, which can be beneficial for human health.

## Figures and Tables

**Table 1:** Experimental scheme.

Control	Fitostim^®^ Alga	Phylgreen^®^
**Phylgreen^®^**	**Control**	**Fitostim^®^ Alga**
**Fitostim^®^ Alga**	**Phylgreen^®^**	**Control**

**Table 2:** Correlation analysis of the studied parameters.

	Dry Weight	Number of Fruits	Fruit Part	Treatment	Harvest Date	Total Pigments	Total Tocopherol	Yield	Color
Dry weight		0.5258	−0.0061	−0.2037	0.5641	0.0193	−0.0304	0.5358	0.0461
Number of fruits			−0.0104	−0.0597	0.7878	0.0648	−0.0346	0.9936	0.2493
Fruit part				−0.0142	−0.0286	0.9709	0.9562	−0.0096	0.9636
Treatment					−0.0175	−0.0049	−0.0418	−0.1389	0.8963
Harvest date						0.0125	−0.1451	0.7895	0.4678
Total pigments							0.9518	0.0613	0.9862
Total tocopherol								−0.0353	0.5377
Yield									0.0579

**Table 3:** Fruit parameters (skin color, dry weight, flesh diameter, and placenta diameter) in algae biostimulant-treated cucumbers.

		8 June 2018 (1st Harvest)			20 June 2018 (2nd Harvest)			4 July 2018 (3rd Harvest)			Average Measured Values		
		Control	Phylgreen^®^	Fitostim^®^ Algae	Control	Phylgreen^®^	Fitostim^®^ Algae	Control	Phylgreen^®^	Fitostim^®^ Algae	Control	Phylgreen^®^	Fitostim^®^ Algae
Skin color	a*	−3.85 ± 0.37 a,^†^	−3.04 ± 0.36 b	−4.03 ± 0.48 a	−3.78 ± 0.51 a	−3.61 ± 0.07 a	−3.63 ± 0.10 a	−4.90 ± 0.20 a	−4.11 ± 0.22 a	−4.44 ± 0.60 a	−4.17 ± 0.24 a	−3.58 ± 0.71 b	−4.03 ± 0.31 a
	b*	5.47 ± 0.68 a	4.32 ± 1.66 b	6.49 ± 0.63 a	5.28 ± 0.96 a	4.72 ± 0.17 a	4.63 ± 0.32 a	7.78 ± 0.53 a	5.74 ± 0.60 c	6.35 ± 1.43 b	6.17 ± 0.22 a	4.92 ± 0.30 b	5.82 ± 0.42 a
	L*	23.48 ± 0.71 a	21.47 ± 2.26 b	20.81 ± 1.53 c	25.12 ± 1.62 a	23.99 ± 0.99 a	24.09 ± 0.50 a	25.11 ± 0.53 a	23.47 ± 0.67 b	23.55 ± 1.04 b	24.56 ± 0.99 a	22.97 ± 1.42 b	22.81 ± 1.22 b
Dry weight		3.47 ± 0.11 b	3.47 ± 0.21 b	3.66 ± 0.17 a	3.89 ± 0.05 a	3.79 ± 0.27 a	3.89 ± 0.31 a	4.27 ± 0.42 a	4.08 ± 0.20 a	3.90 ± 0.27 a	3.87 ± 0.11 a	3.78 ± 0.15 a	3.81 ± 0.13 a
Flesh diameter		27.04 ± 0.66 a	26.46 ± 0.67 a	26.09 ± 0.49 a	29.08 ± 0.37 a	26.90 ± 0.33 a	29.53 ± 0.39 a	29.49 ± 1.28 a	28.60 ± 1.00 a	27.00 ± 1.23 a	28.53 ± 1.04 a	27.32 ± 0.99 a	27.54 ± 1.23 a
Placenta diameter		22.50 ± 1.20 a	21.56 ± 1.10 a	21.71 ± 0.95 a	21.18 ± 1.04 a	21.02 ± 1.31 a	20.76 ± 0.39 a	20.44 ± 1.65 a	20.84 ± 1.20 a	20.07 ± 1.05 a	21.37 ± 2.44 a	21.14 ± 1.99 a	20.84 ± 2.05 a

^†^ Data are means ± standard error (n = 5). Data with different lowercase letters (a to c) among treatments (each column) are significantly different, within the same harvest dates (HSD test; *p* < 0.05).

## Data Availability

The data presented in this study are available on request from the corresponding author. The data are not publicly available due to privacy.

## Supplementary Materials

The following supporting information can be downloaded at: https://www.mdpi.com/article/10.3390/foods13030401/s1. Table S1: Individual/total pigments and tocopherols in cucumber flesh picked at three harvest times; Table S2: Individual/total pigments and tocopherols in cucumber skin picked at three harvest times.
