# Supplementary material for: Impact of Two Brown Seaweed (Ascophyllum nodosum L.) Biostimulants on the Quantity and Quality of Yield in Cucumber (Cucumis sativus L.)
Source: Foods. 2024 Jan 26;13(3):401. doi: 10.3390/foods13030401 (PMC10855078; doi:10.3390/foods13030401)
Supplement: Supplementary file 1 [file foods-13-00401-s001.zip › foods-2811208-supplementary.pdf]

Table S1. Individual/total pigments and tocopherols in cucumber flesh picked at three harvest times.

Flesh	8. Jun. 2018 (1st harvest)			20. Jun. 2018 (2nd harvest)			4. Jul. 2018 (3rd harvest)			Average total metabolites		
	Control	Phylgreen®	Fitostim® algae	Control	Phylgreen®	Fitostim® algae	Control	Phylgreen®	Fitostim® algae	Control	Phylgreen®	Fitostim® algae
Neoxanthin	7.75 ± 0.70 b⸙	11.33 ± 1.48 a	12.26 ± 1.48 a	10.98 ± 0.68 a	11.07 ± 0.82 a	8.81 ± 0.70 a	14.66 ± 2.06 a	12.42 ± 1.23 a	13.38 ± 1.94 a	11.13 ± 1.15 a	11.61 ± 1.18 a	11.48 ± 1.37 a
Violaxanthin	4.91 ± 0.64 c	8.47 ± 0.47 b	10.03 ± 0.53 a	7.63 ± 0.52 a	6.75 ± 0.33 a	5.94 ± 0.35 a	9.86 ± 0.70 a	9.47 ± 0.44 a	10.53 ± 1.11 a	7.47 ± 0.62 b	8.23 ± 0.42 a	8.83 ± 0.66 a
Antheraxanthin	6.97 ± 0.57 c	11.06 ± 1.34 b	13.22 ± 1.02 a	9.94 ± 0.59 a	9.77 ± 0.42 a	8.09 ± 0.74 a	12.80 ± 1.39 a	11.06 ± 0.78 a	10.91 ± 1.31 a	9.90 ± 0.85 b	10.63 ± 0.84 a	10.74 ± 1.02 a
Lutein	20.83 ± 2.80 b	25.09 ± 3.51 a	25.63 ± 2.62 a	19.44 ± 0.93 a	21.68 ± 1.51 a	15.81 ± 1.12 b	25.46 ± 2.90 a	21.19 ± 3.11 b	24.34 ± 4.27 a	21.91 ± 2.21 a	22.65 ± 2.71 a	21.93 ± 2.67 a
Chlorophyll b	53.40 ± 6.08 c	66.36 ± 8.92 b	76.16 ± 3.94 a	41.20 ± 2.82 b	47.36 ± 4.02 a	32.18 ± 2.20 c	52.12 ± 7.61 a	46.28 ± 8.43 b	53.66 ± 12.77 a	48.91 ± 5.50 b	53.33 ± 7.13 a	54.00 ± 6.30 a
Chlorophyll a	161.80 ± 17.56 c	188.81 ± 23.76 b	203.49 ± 19.57 a	138.19 ± 9.31 b	157.09 ± 11.81 a	102.07 ± 7.11 c	171.41 ± 20.37 a	151.54 ± 25.51 b	168.87 ± 30.75 a	157.13 ± 15.75 b	165.82 ± 20.36 a	158.15 ± 19.14 b
β-carotene	9.12 ± 0.56 b	11.46 ± 1.39 a	11.58 ± 1.11 a	10.16 ± 0.48 a	10.55 ± 0.45 a	8.14 ± 0.62 b	12.86 ± 1.10 a	11.46 ± 1.23 a	12.19 ± 1.44 a	10.71 ± 0.71 a	11.16 ± 1.02 a	10.64 ± 1.05 a
**TAP**	**264.78 ± 10.52 b**	**322.58 ± 15.62 a**	**352.37 ± 16.73 a**	**237.54 ± 13.67 b**	**264.27 ± 12.78 a**	**181.04 ± 10.85 c**	**298.90 ± 17.73 a**	**263.42 ± 16.74 b**	**293.88 ± 10.08 a**	**267.16 ± 10.73 b**	**283.42 ± 11.47 a**	**275.77 ± 15.78 a**
Γ-tocopherol	0.42 ± 0.09 b	0.54 ± 0.05 a	0.58 ± 0.06 a	0.23 ± 0.06 b	0.29 ± 0.08 a	0.15 ± 0.06 c	0.43 ± 0.07 a	0.35 ± 0.07 b	0.41 ± 0.03 b	0.36 ± 0.07 a	0.39 ± 0.07 a	0.38 ± 0.07 a
α-tocopherol	6.99 ± 0.81 a	7.72 ± 0.59 a	4.67 ± 1.60 b	4.65 ± 1.06 b	7.29 ± 0.88 a	5.26 ± 0.84 b	7.52 ± 0.55 a	6.93 ± 0.50 a	3.93 ± 1.12 b	6.39 ± 0.63 a	7.31 ± 0.55 a	4.62 ± 0.36 b
**TAT**	**7.41 ± 0.62 b**	**8.26 ± 0.13 a**	**5.25 ± 0.55 c**	**4.88 ± 0.42 b**	**7.58 ± 0.73 a**	**5.41 ± 0.33 b**	**7.95 ± 0.90 a**	**7.28 ± 0.72 a**	**4.34 ± 0.31 b**	**6.75 ± 0.57 a**	**7.71 ± 0.74 a**	**5.00 ± 0.33 b**

⸙ Data are means ± standard error (n = 5). Data with different lower-case letters (a to c) for each individual substance, among treatments are significantly different, within the same harvest dates (HSD test; $p < 0.05$). TAP = Total analyzed pigments; TAT = Total analyzed tocopherols.

Table S2. Individual/total pigments and tocopherols in cucumber skin picked at three harvest times.

Skin	8. Jun. 2018 (1st harvest)			20. Jun. 2018 (2nd harvest)			4. Jul. 2018 (3rd harvest)			Average total metabolites		
	Control	Phylgreen®	Fitostim® algae	Control	Phylgreen®	Fitostim® algae	Control	Phylgreen®	Fitostim® algae	Control	Phylgreen®	Fitostim® algae
neoxanthin	348.95 ± 19.68 a[ǂ]	269.71 ± 14.57 b	324.02 ± 37.25 a	240.80 ± 10.94 a	253.85 ± 28.62 a	236.23 ± 23.44 a	265.77 ± 14.94 b	251.15 ± 10.29 b	292.78 ± 33.96 a	285.17 ± 15.19 a	258.24 ± 17.83 b	284.34 ± 31.55 a
violaxanthin	165.93 ± 8.17 a	140.60 ± 8.52 b	173.10 ± 13.64 a	127.06 ± 3.49 a	128.96 ± 11.27 a	125.16 ± 10.73 a	131.43 ± 10.05 b	132.33 ± 10.59 b	160.44 ± 10.22 a	141.47 ± 7.24 a	133.96 ± 10.13 b	152.90 ± 11.53 a
antheraxanthin	136.90 ± 7.89 a	113.76 ± 5.70 b	143.56 ± 11.79 a	123.30 ± 3.60 a	127.74 ± 6.52 a	116.67 ± 9.49 a	147.72 ± 9.59 b	142.99 ± 6.81 b	160.01 ± 6.74 a	135.97 ± 7.03 a	128.16 ± 6.34 a	140.08 ± 9.34 a
lutein	1019.28 ± 37.94 a	798.23 ± 33.24 c	903.49 ± 77.84 b	783.47 ± 32.88 b	899.71 ± 61.75 a	819.92 ± 79.58 a	954.53 ± 54.50 b	908.67 ± 30.89 b	1038.63 ± 57.09 a	919.09 ± 41.77 a	868.87 ± 41.96 b	920.68 ± 71.51 a
chlorophyll b	2092.67 ± 98.76 a	1583.13 ± 49.86 c	1862.57 ± 168.58 b	1590.76 ± 98.79 b	1860.28 ± 169.69 a	1667.88 ± 163.09 b	1961.78 ± 147.18 b	1917.63 ± 110.02 b	2303.72 ± 127.25 a	1881.73 ± 114.91 a	1787.01 ± 109.86 b	1944.73 ± 152.97 a
chlorophyll a	2838.37 ± 152.82 a	2244.85 ± 43.71 c	2617.46 ± 230.73 b	2337.33 ± 167.43 b	2733.88 ± 235.03 a	2390.85 ± 212.17 b	2897.10 ± 198.54 b	2758.34 ± 177.74 b	3228.38 ± 168.55 a	2690.93 ± 172.93 a	2579.02 ± 152.16 a	2745.57 ± 203.82 a
β-carotene	370.00 ± 9.46 a	341.97 ± 12.54 a	354.32 ± 22.00 a	322.51 ± 3.77 a	352.10 ± 10.08 a	314.14 ± 27.59 a	356.89 ± 15.53 a	356.11 ± 9.62 a	353.95 ± 20.92 a	349.80 ± 9.58 a	350.06 ± 10.75 a	340.80 ± 23.50 a
**TAP**	**7008.40 ± 173.83 a**	**5367.25 ± 100.51 c**	**6378.52 ± 153.52 b**	**5525.23 ± 144.06 b**	**6356.52 ± 129.56 a**	**5670.85 ± 200.01 b**	**6715.22 ± 196.77 b**	**6467.22 ± 182.72 b**	**7537.91 ± 165.62 a**	**6404.18 ± 177.83 a**	**6105.33 ± 171.06 b**	**6529.09 ± 206.79 a**
δ-tocopherol	3.31 ± 0.85 b	3.32 ± 0.17 b	4.59 ± 0.28 a	3.20 ± 0.15 a	2.87 ± 0.31 a	3.40 ± 0.22 a	2.81 ± 0.24 b	3.32 ± 0.62 a	3.83 ± 0.24 a	3.11 ± 0.41 b	3.17 ± 0.37 b	3.94 ± 0.25 a
Γ-tocopherol	8.35 ± 0.88 a	8.16 ± 0.26 a	9.60 ± 0.58 a	7.50 ± 0.34 a	6.94 ± 0.43 a	7.55 ± 1.64 a	9.18 ± 0.28 a	9.27 ± 0.49 a	9.05 ± 0.48 a	8.34 ± 0.50 a	8.12 ± 0.39 a	8.73 ± 0.90 a
α-tocopherol	84.02 ± 3.05 a	64.29 ± 3.96 c	72.06 ± 4.17 b	52.00 ± 1.21 a	55.23 ± 4.54 a	49.80 ± 1.29 a	53.20 ± 5.41 a	55.47 ± 5.07 a	54.80 ± 1.75 a	63.07 ± 3.22 a	58.33 ± 4.52 a	58.89 ± 2.41 a
**TAT**	**95.68 ± 7.37 a**	**75.77 ± 4.70 c**	**86.25 ± 6.36 b**	**62.70 ± 5.72 a**	**65.04 ± 6.21 a**	**60.75 ± 4.55 a**	**65.19 ± 3.78 a**	**68.06 ± 5.36 a**	**67.68 ± 5.22 a**	**74.52 ± 6.53 a**	**69.62 ± 3.52 a**	**71.56 ± 4.99 a**

ǂ Data are means ± standard error (n = 5). Data with different lower-case letters (a to c) for each individual substance among treatments are significantly different within the same harvest dates (HSD test; $p < 0.05$). TAP = Total analyzed pigments; TAT = total analyzed tocopherols.
